# Supplementary material for: An open-source framework for large-scale, flexible evaluation of biomedical text mining systems
Source: J Biomed Discov Collab. 2008 Jan 29;3:1. doi: 10.1186/1747-5333-3-1 (PMC2276192; doi:10.1186/1747-5333-3-1)
Supplement: Additional file 1 — Evaluation platform source code. The additional file contains the software evaluation framework discussed in this paper. The most current release can be downloaded from SourceForge.net [1]. See the accompanying README file for instructions on installation and use. Also included in the distribution is a collection of UIMA wrappers for some commonly used BioNLP tools and annotated corpora. [file 1747-5333-3-1-S1.zip › bionlp_uima_repository-1.0.1/docs/api/src-html/edu/uchsc/ccp/uima/ae/util/converter/DocumentLevelAnnotationCreator_AE.html]

```
001    package edu.uchsc.ccp.uima.ae.util.converter;
002    
003    import java.util.Arrays;
004    import java.util.HashSet;
005    import java.util.List;
006    import java.util.Set;
007    
008    import org.apache.uima.analysis_engine.ResultSpecification;
009    import org.apache.uima.analysis_engine.annotator.AnnotatorConfigurationException;
010    import org.apache.uima.analysis_engine.annotator.AnnotatorContext;
011    import org.apache.uima.analysis_engine.annotator.AnnotatorContextException;
012    import org.apache.uima.analysis_engine.annotator.AnnotatorInitializationException;
013    import org.apache.uima.analysis_engine.annotator.AnnotatorProcessException;
014    import org.apache.uima.analysis_engine.annotator.JTextAnnotator_ImplBase;
015    import org.apache.uima.jcas.JCas;
016    
017    import edu.uchsc.ccp.uima.util.UIMA_Util;
018    import edu.uchsc.ccp.util.nlp.annotation.TextAnnotation;
019    import edu.uchsc.ccp.util.nlp.annotation.comparison.DocumentLevelAnnotationCreator;
020    
021    /**
022     * creates document-level annotations of the specified type. The user can optionally stipulate that certain slots be present in order for the
023     * annotation to be created as a document-level annotation. One example for this would be to create document-level annotations of proteins, and
024     * require that the proteins have an entrez_gene_id slot. This way, the gene name normalizer can be tested using our existing comparison machinery.
025     * 
026     * @author williamb
027     * 
028     */
029    public class DocumentLevelAnnotationCreator_AE extends JTextAnnotator_ImplBase {
030    
031        public static final String PARAM_ANNOTATION_TYPE_TO_CONVERT = "AnnotationTypeToConvert";
032    
033        public static final String PARAM_REQUIRED_SLOTS = "RequiredSlots";
034    
035        public static final String PARAM_ANNOTATORS_TO_IGNORE = "AnnotatorsToIgnore";
036    
037        private Set<String> requiredSlots;
038    
039        private String annotationTypeToConvert;
040    
041        private DocumentLevelAnnotationCreator documentLevelAnnotationCreator;
042    
043        private Set<Integer> annotatorIDsToIgnore;
044    
045        @Override
046        public void initialize(AnnotatorContext ac) throws AnnotatorInitializationException, AnnotatorConfigurationException {
047            /* read in input parameters, and initialize a list of annotator ids to keep */
048            try {
049                requiredSlots = new HashSet<String>(Arrays.asList((String[]) ac.getConfigParameterValue(PARAM_REQUIRED_SLOTS)));
050                annotationTypeToConvert = (String) ac.getConfigParameterValue(PARAM_ANNOTATION_TYPE_TO_CONVERT);
051                try {
052                    annotatorIDsToIgnore = new HashSet<Integer>(Arrays.asList((Integer[]) ac.getConfigParameterValue(PARAM_ANNOTATORS_TO_IGNORE)));
053                } catch (NullPointerException npe) {
054                    annotatorIDsToIgnore = new HashSet<Integer>();
055                }
056            } catch (AnnotatorContextException e) {
057                e.printStackTrace();
058            }
059    
060            documentLevelAnnotationCreator = new DocumentLevelAnnotationCreator(annotationTypeToConvert, requiredSlots);
061    
062            super.initialize(ac);
063        }
064    
065        /**
066         * cycle through all annotations and convert the appropriate ones to document-level annotations
067         */
068        public void process(JCas jcas, ResultSpecification arg1) throws AnnotatorProcessException {
069            UIMA_Util uimaUtil = new UIMA_Util();
070            List<TextAnnotation> annotationsInCas = uimaUtil.getTextAnnotationsFromJCas(jcas);
071    
072            List<TextAnnotation> documentLevelAnnotations = documentLevelAnnotationCreator.createDocumentLevelAnnotations(annotationsInCas,
073                    annotatorIDsToIgnore);
074    
075            uimaUtil.putTextAnnotationsIntoJCas(jcas, documentLevelAnnotations);
076        }
077    
078    }
```
